# Interleukin-4 enhances proliferation of human pancreatic cancer cells: evidence for autocrine and paracrine actions

**DOI:** 10.1038/sj.bjc.6602416

**Published:** 2005-02-15

**Authors:** O Prokopchuk, Y Liu, D Henne-Bruns, M Kornmann

**Affiliations:** 1 Department of Visceral and Transplantation Surgery, University of Ulm, Steinhoevelstrasse 9, 89075 Ulm, Germany; 2 Department of Internal Medicine II, Section of Sports and Rehabilitation Medicine, University of Ulm, Steinhoevelstrasse 9, 89075 Ulm, Germany

**Keywords:** cytokine, growth factor, mitogenic signalling, interleukin, pancreatic cancer

## Abstract

Interleukin-4 (IL-4) is an immunomodulatory cytokine, which can inhibit the growth of tumour cells. Pancreatic cancer cells and tissues express high levels of IL-4 receptors. The aim of this study was to characterise the effects of IL-4 on the growth and signalling pathways of pancreatic cancer cells. Cell growth was determined by cell counting and MTT assays in association with fluorescence-activated cell sorter analysis, IL-4 expression using ELISA and real-time PCR techniques, and signal transduction using immunoprecipitation or immunoblot analysis. We now report for the first time that IL-4 significantly enhanced the growth of five out of six cultured pancreatic cancer cell lines in a dose-dependent manner in association with an increased fraction of cells in S-phase. Surprisingly, all six cell lines expressed endogenous IL-4, and IL-4 was detectable in the supernatant. Incubating cells with neutralising IL-4 antibodies resulted in a significant inhibition of basal growth in three cell lines, including IL-4-unresponsive MIA PaCa-2 cells, which however expressed the highest endogenous IL-4 levels. Interleukin-4 enhanced activity of MAPK, Akt-1, and Stat3 in IL-4-responsive, but not in IL-4-unresponsive MIA PaCa-2 cells; however, IL-4 enhanced tyrosine phosphorylation of insulin receptor substrate-1 and -2 in all cell lines. Our results demonstrate for the first time that pancreatic cancer cells produce IL-4 and that IL-4 can act as a growth factor in pancreatic cancer cells. Together with the observation that neutralising IL-4 antibodies can inhibit the growth of these cells, our results suggest that IL-4 may act as an autocrine growth factor in pancreatic cancer cells and also give rise to the possibility that cancer-derived IL-4 may suppress cancer-directed immunosurveillance *in vivo* in addition to its growth-promoting effects, thereby facilitating pancreatic tumour growth and metastasis.

Interleukin-4 (IL-4) is a secreted, potentially glycosylated anti-inflammatory and immunomodulatory 18 kDa cytokine with pleiotropic functions sharing several biologic activities with IL-13. It is produced mainly by a subpopulation of activated T-cells, TH2 CD4(+) helper cells, and promotes the proliferation and differentiation of activated B-cells, the expression of class II MHC antigens, and of low-affinity IgE receptors in resting B-cells (Paul, 1991).

The biological activities of IL-4 are mediated by a specific IL-4 receptor (IL-4R) that is expressed at densities of 100–5000 copies cell^−1^ (Nelms *et al*, 1999). IL-4 receptor *α* (IL-4R*α*) was found to be expressed on solid human tumours, including malignant melanoma, breast carcinoma, ovarian carcinoma, mesothelioma, glioblastoma, renal cell carcinoma, head and neck carcinoma, and AIDS-associated Kaposi's sarcoma (Kawakami *et al*, 2001; Gooch *et al*, 2002). It was also reported that several cultured pancreatic cancer cell lines express IL-4R*α* (Kornmann *et al*, 1999a). Using binding assays, it could be demonstrated that PANC-1 pancreatic cancer cells express high numbers of specific IL-4 binding sites (Kawakami *et al*, 2002). The significance of IL-4Rs expression in pancreatic cancer and other solid tumours is still unknown. In contrast to its growth stimulatory effect on lymphocytes, IL-4 has been shown to have a modest but direct inhibitory effect on the growth of tumour cells of haematopoietic and nonhaematopoietic origin *in vitro* and *in vivo*, including those derived from human melanoma, non-Hodgkin's malignant B-lymphoma, and colon, renal, gastric, and breast carcinoma (Defrance *et al*, 1992; Gallagher and Zaloom, 1992; Morisaki *et al*, 1992; Toi *et al*, 1992, Obiri *et al*, 1993).

The different effects of IL-4 on cell proliferation presently remain unclear. Chang and co-workers proposed that binding of IL-4 to its receptor can trigger at least three signalling pathways including insulin receptor substrate (IRS)/signal transducer and activator of transcription 3 (Stat3), Stat6, and Stat1 activation. Whereas the activation of IRS substrates and Stat3 may result in cell proliferation, activation of Stat6 seems to be involved in regulation of the immune system such as T helper cell differentiation and IgE class switching (Chang *et al*, 2000; Scholz *et al*, 2003). In contrast, IL-4-mediated activation of Stat1 results in cell growth inhibition (Chang *et al*, 2000).

Pancreatic cancer is a deadly disease with a poor prognosis and a propensity to rapidly metastasise to tissues and lymph nodes while exhibiting resistance to cancer-directed immune mechanisms. Pancreatic cancers not only overexpress multiple growth factors including members of the insulin-like growth factor (IGF) family like IGF-I (Korc, 1998), but also overexpress the intracellular signal transducers of this pathway, IRS-1 and IRS-2 (Kornmann *et al*, 1998). Pancreatic cancer cells and tissues also express high levels of IL-4R*α* (Kornmann *et al*, 1999a, 1999b; Kawakami *et al*, 2002).

In view of the abundant production of IL-4 by activated T-lymphocytes, the proclivity of pancreatic cancer to metastasise and to avoid cancer-directed immune mechanisms, this project aimed to characterise the effects of IL-4 and neutralising IL-4 antibodies on the growth of cultured human pancreatic cancer cells, the expression and secretion of IL-4 by human pancreatic cancer cells, and IL-4-dependent signalling pathways.

## MATERIALS AND METHODS

### Materials

The Cycle Test Plus DNA Reagent Kit for fluorescence-activated cell sorter (FACS) analysis was purchased from Becton Dickinson Immunocytometry Systems (San Jose, CA, USA). Human recombinant IL-4 and IGF-I, 3-(4,5-methylthiazol-2-yl)-2,5-diaphenyltertrazolium bromide (MTT), monoclonal mouse anti-*β*-actin antibody, and secondary horseradish-conjugated anti-rabbit were from Sigma Chemical Co. (St Louis, MO, USA). Rabbit polyclonal anti-phospho-Akt antibody (Ser 473)-R (1 : 500), mouse monoclonal Akt-1 (B-1) antibody (1 : 500), anti-active mitogen-activated protein kinase (MAPK) (p-ERK, E-4) mouse monoclonal antibody (1 : 5000), ERK-2 (C-14) rabbit polyclonal antibody (1 : 2000), mouse monoclonal IgG_1_ antibody p-Stat1 (A-2) (1 : 200), rabbit polyclonal antibody Stat1 p84/p91 (E-23) (1 : 250), mouse polyclonal antibody p-Stat3 (B-7) (1 : 1000), rabbit polyclonal antibody Stat3 (K-15) (1 : 200), rabbit polyclonal antibody Stat6 (1 : 1000), mouse monoclonal IgG_2b_ antibody p-Tyr (PY99) (1 : 2000), and mouse whole-cell lysate from normal embryo fibroblast cells (NIH/3T3 cells) were from Santa Cruz Biotechnology (Santa Cruz, CA, USA). Monoclonal anti-human IL-4 antibody and mouse monoclonal anti-human CD5 antibody were obtained from R&D Systems (Minneapolis, MN, USA). The human IL-4 immunoassay kit was from BioSource International Inc. (Camarillo, CA, USA). Rabbit immunoaffinity-purified anti-rat IRS-1 (1 : 2000) and rabbit polyclonal anti-mouse IRS-2 (1 : 2000) were from Upstate Biotechnology Inc. (Lake Placid, NY, USA). The RNeasy Mini Kit for RNA extraction was from Qiagen GmbH (Hilden, Germany). SuperScript First-Strand Synthesis System for RT–PCR was from Invitrogen (Carlsbad, CA, USA). The Real–Time PCR Kit for Human IL-4 Gene Expression was from Maxim Biotech. Inc. (CA, USA). ASPC-1, CAPAN-1, MIA PaCa-2, and PANC-1 human pancreatic cancer cells were purchased from American Type Culture Collection (ATCC, Rockville, MD, USA). COLO-357 and T3M4 human pancreatic cancer cells were a gift from RS Metzgar (Duke University, Durham, NC, USA).

### Cell culture

COLO-357, MIA PaCa-2, and PANC-1 cells were grown in DME medium, whereas ASPC-1, CAPAN-1, and T3M4 cells were grown in RPMI-1640 medium. All media were supplemented with 8% FBS, penicillin G (100 U ml^−1^), and streptomycin (100 *μ*g ml^−1^), termed complete medium. Cells were maintained at 37°C in humidified air with 5% CO_2_. Medium containing antibiotics, 0.1% BSA, 5 mg l^−1^ transferrin, and 5 *μ*g l^−1^ selenious acid was termed serum-free medium and used when indicated for the specific assays.

### Growth assays

For cell counting, cells (150 000 well^−1^) were seeded in 12-well plates in complete medium (3 ml well^−1^). After 24 h, the medium was replaced by serum-free medium (500 *μ*l well^−1^) for the indicated times in the absence or presence of IL-4 (5 nM) or IGF-I (5 nM). The medium was changed daily, including the respective additions. For cell counting, cells were trypsinised, resuspended, adjusted to 1 ml, and counted under a light microscope using a haemocytometer.

Cell growth was also determined by the MTT assay as described (Kornmann *et al*, 1998). Cells (10 000 well^−1^) were seeded in 96-well plates, incubated for 24 h in complete medium, and then for 48 h in serum-free medium in the absence or presence of the indicated substrates. The assay was also performed in the presence of 8% FBS. In these cases, only 5000 cells well^−1^ were seeded and IL-4 was added after 24 h for 48 h. To initiate the assay, MTT reagent was added and cells were incubated for an additional 4 h at 37°C. After removal of the medium and dissolving the crystals with acidified isopropanol, the samples were analysed using an ELISA plate reader at 570 nm. The value at 650 nm was subtracted as background.

### Cell cycle analysis: fluorescence-activated cell sorter

To determine the effect of IL-4 and IGF-I on cell cycle, cells were seeded in six-well plates, incubated for 24 h in complete medium to 60% confluency, and then serum starved for 24 h. The medium was replaced by serum-free medium in the absence or presence of IL-4 (5 nM) or IGF-I (5 nM) for another 24 h. Thereafter, FACS analysis was performed using a CycleTest Plus kit according to the instructions of the manufacturer and FACScan (Becton Dickinson) analysis system equipped with a FACStation, MAC PowerPC computer, and CellQuest acquisition software as previously described (Kornmann *et al*, 1999a, 1999b).

### Interleukin-4 ELISA

Total cell lysates were prepared as described (Kornmann *et al*, 1998). After adjusting the protein concentration using lysis buffer, 100 *μ*l aliquots were used for the IL-4 sandwich ELISA carried out according to the protocol of the manufacturer. To detect IL-4 in the supernatant, cells were cultured in six-well plates to 70% confluency. After washing, cells were cultured in 1 ml of serum-free medium including proteinase inhibitors (Kornmann *et al*, 1998) for 48 h. The medium was then harvested and centrifuged before subjecting 100 *μ*l aliquots to the ELISA. Cells were counted to determine the amount of IL-4 secreted per cell. In the present study, the interassay variability was less than 28% and the intra-assay variability less than 5%.

### Analysis of IL-4 mRNA

Exponentially growing cells were harvested and pelleted by centrifugation at 300 **g** for 5 min after washing twice with PBS. Total RNA extraction and reverse transcription was then performed according to the instructions of the manufacturer.

The design of the IL-4 5′ and 3′ primers was based on the sequence with GenBank accession number NM_000589. The design of *β*-actin 5′ and 3′ primers was based on the sequence with GenBank accession number NM_001101. Primer sets for IL-4 and FRET probes for IL-4 with known cDNA copy number were designed to generate an 81 bp PCR product as described by the manufacturer (Maxim Biotech Inc., CA, USA). Primers for *β*-actin were sense GGCATCCTCACCCTGAAGTA and antisense GTCAGGCAGCTCGTAGCTCT resulting in a 525 bp PCR product.

Real-time RT–PCR amplification was performed using the QuantiTect™ SYBR Green PCR Kit (QIAGEN, Germany). Prior to quantitative analysis, several titration experiments, for MgCl_2_, and efficiency tests were performed to determine optimum amplification conditions. Standard curves containing a specific number of cDNA copies were generated for the IL-4 gene transcript. After the PCR had been completed, the LightCycler software (Roche Diagnostics, Mannheim, Germany) automatically converted the raw data into copies of target molecules using standard curves.

In brief, the total reaction volume for each sample was 20 *μ*l and contained 2 *μ*l cDNA solution, 10 pmol of each primer, 100 *μ*M of each 2-deoxynucleotide 5′-triphosphate, 4 mM MgCl_2_, and 1 U of *Taq* polymerase and Syber Green as indicator. After hot activation at 94°C for 15 min, 55 cycles of 15 s denaturation at 94°C, 25 s annealing at 68°C, and 16 s synthesis at 72°C were performed. The colour signal (Syber Green, which combines exclusively to the double-stranded DNA) was measured in a real-time model at the end of the DNA synthesis phase, and the crossing point (where the colour signal began to increase exponentially) was calculated. The specificity of DNA amplification was examined with analysis of the melting curve. Samples with known cDNA copy number were used as a positive control for IL-4 PCR, and no RT cDNA samples and samples without cDNA (water) served as negative controls.

### Immunoblot analysis

Cells were cultured to 80% confluency and then serum starved for 18 h prior to the analysis including growth factors followed by immunoblot analysis with the respective primary and secondary antibodies as described (Kornmann *et al*, 1999a, 1999b). To confirm an equal transfer of the proteins to the membranes, stripping of the membranes was performed when indicated using 50 ml of stripping buffer for 30 min in a water bath at 50°C (Kornmann *et al*, 1999b).

### Immunoprecipitation

Insulin receptor substrate immunoprecipitation assays were carried out as previously described (Kornmann *et al*, 1998). Cells were grown to 80% confluency and serum starved for 18 h before the respective treatment. After cell lysis using a modified RIPA buffer (Kornmann *et al*, 1998), lysates (1000 *μ*g 1000 *μ*l^−1^) were incubated for 2 h at room temperature with 4 *μ*g of the respective antibody (4 *μ*g IRS-1 or 4 *μ*g IRS-2) on a rotator followed by 45 min incubation after adding 50 *μ*l of slurry protein A sepharose. For subsequent antiphosphotyrosine immunoblot analysis, precipitates were washed three times with ice-cold PBS, resuspended in 50 *μ*l of 2 × Laemmli buffer, and boiled for 5 min at 100°C before the analysis. Desired volumes (20 *μ*l) were transferred to PAGE–SDS minigels followed by immunoblot analysis.

### Statistics

Results are expressed as mean±s.e.m. or mean±s.d. When indicated, Student's *t*-test or Mann–Whitney rank sum test was used for statistical analysis using SigmaStat statistical software. *P*<0.05 was taken as the level of significance (two-sided).

## RESULTS

### Effects of IL-4 on pancreatic cancer cell proliferation

**Figure 1:**
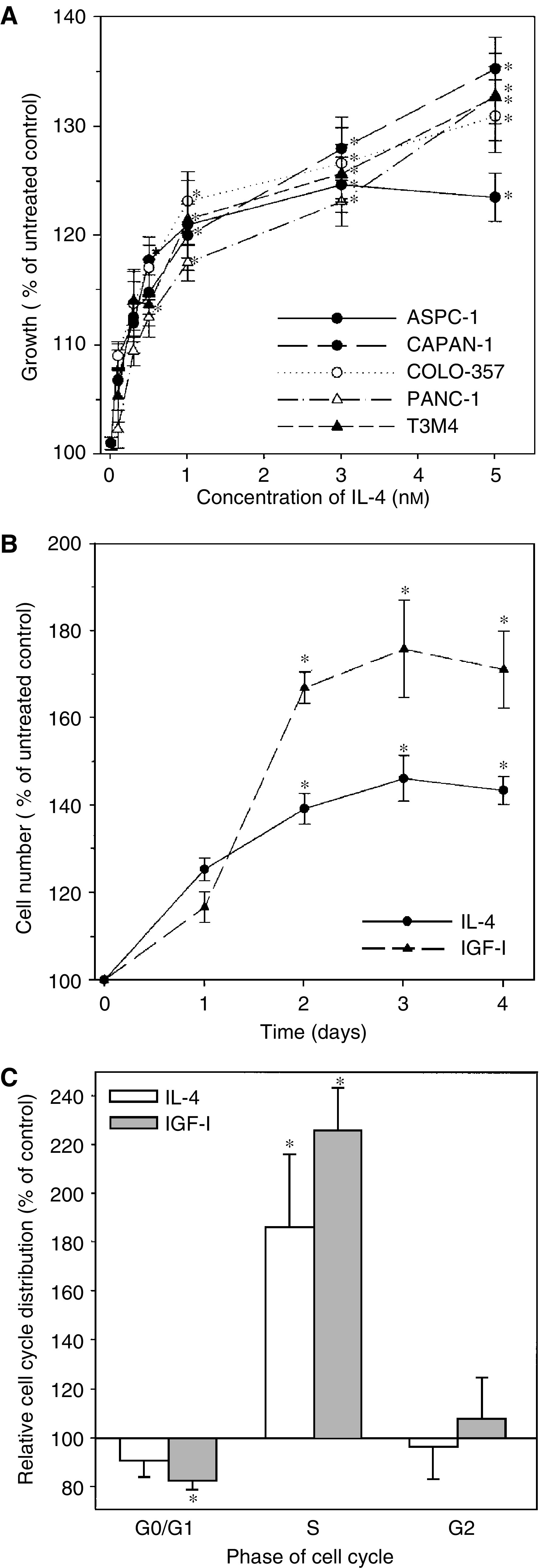
Effect of IL-4 on pancreatic cancer cell growth and cell cycle distribution. (**A**) IL-4 dose–response. Indicated cells were cultured in 96-well plates in the absence and presence of increasing concentrations of IL-4. Cell growth was determined by the MTT assay after 48 h. Results are expressed as growth (in %) of the corresponding untreated controls and are the means (±s.e.m.) of quadruplicate determinations from at least three separate experiments. (**B**) IL-4 and IGF-I time course. PANC-1 cells were cultured in the absence and presence of 5 nM IL-4 (solid line, circle) or 5 nM IGF-I (long dash line, triangle) for the indicated times. Cell growth was determined by cell counting. Results are expressed as cell number (in %) of untreated controls and are means (±s.e.m.) of triplicate determinations from at least three separate experiments. (**C**) Effect of IL-4 and IGF-I on cell cycle distribution. PANC-1 cells were cultured in the absence or presence of 5 nM IL-4 (open bars) or 5 nM IGF-I (hatched bars) for 24 h followed by FACS analysis. Results are shown as relative difference of cell cycle fractions in % compared to the corresponding untreated controls and are means (±s.d.) of at least five separate experiments. ^*^P<0.05 compared to untreated controls.

Human IL-4 exerted a dose-dependent increase on the growth of five pancreatic cancer cell lines under serum-free conditions (Figure 1A). Neither an increase of the IL-4 concentration to 10 nM nor a prolongation of the incubation period to 72 h results in an enhanced proliferation compared to 5 nM IL-4 for 48 h (data not shown). Interleukin-4 (5 nM) did not alter the growth of MIA PaCa-2 cells (5±2.4% s.e.m.). IL-4 (5 nM) enhanced the growth of ASPC-1 and COLO-357 cells by 31% (±7.5% s.e.m.) and 16% (±1.3% s.e.m.), respectively, under similar conditions in the presence of 10% serum (*P*<0.02). The growth of CAPAN-1 (25±9.8% s.e.m.) and PANC-1 (14±2.2% s.e.m.) was also enhanced in the presence of 10% serum, but these effects were not significant (*n*=3). Similar to the serum-free conditions, the growth of MIA PaCa-2 cells was not altered by IL-4 (5 nM) in the presence of 10% serum (−8.7±4.9% s.e.m.).

To confirm the results obtained by MTT assay suggesting that IL-4 can act like a growth factor in pancreatic cancer cells, cell counting and comparison with the growth-promoting effects of IGF-I was performed next in PANC-1 cells. Interleukin-4 (5 nM) resulted in an increase in cell number in PANC-1 during the whole 4-day incubation period. The maximal increase in proliferation for IL-4 was observed on day 3, resulting in a 39% (±3.5% s.e.m.) increase in cell number (Figure 1B). For IGF-I, a well-known potent mitogenic growth factor, a maximal increase in cell number was observed also on day 3, resulting in a 76% (±11% s.e.m.) increase in cell number (Figure 1B). Flow cytometric analysis revealed that the enhanced proliferation of PANC-1 cells in the presence of IL-4 was associated with a marked increase in the number of cells in S phase comparable to IGF-I (Figure 1C).

### Expression of IL-4 in human pancreatic cell lines

**Figure 2:**
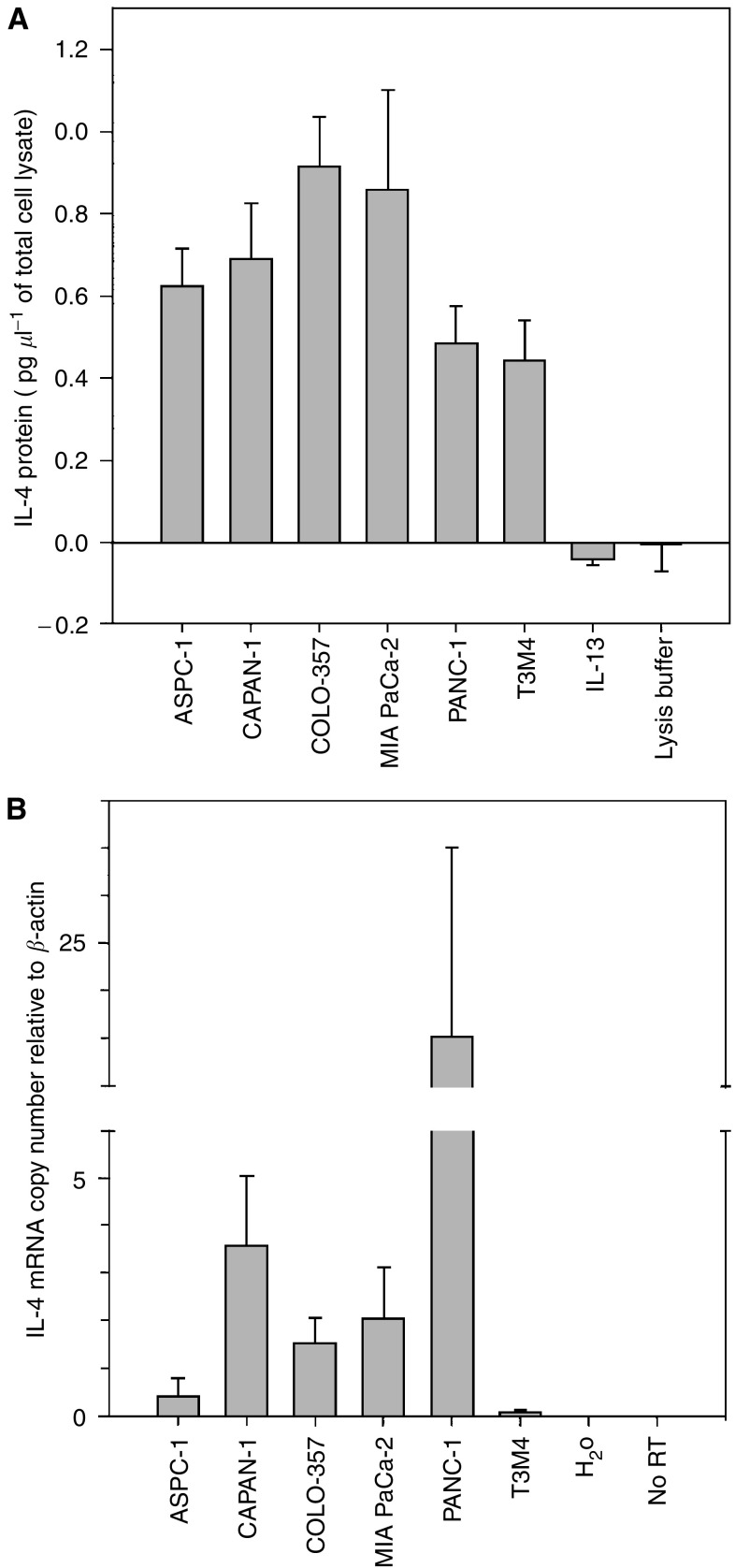
Interleukin-4 expression in pancreatic cancer cells. (**A**) Interleukin-4 ELISA. Total cell lysates (100 *μ*g) of indicated cells were subjected to IL-4 sandwich ELISA. Results are shown as IL-4 protein concentration per *μ*g of the corresponding total cell lysate and are means (±s.d.) of three separate determinations. IL-13 (500 ng) and lysis buffer alone were used as negative controls. (**B**) Interleukin-4 real-time RT–PCR. After reverse transcription, IL-4-specific real-time PCR was performed using *β*-actin as the housekeeping gene. Results are shown as IL-4 mRNA copy number normalised to *β*-actin and are means (±s.d.) of three separate determinations. No RT samples (PANC-1) and water alone served as negative controls.

In view of the mitogenic effects of IL-4, we next sought to determine whether these cells express this cytokine. Determined by ELISA, the IL-4 protein was detectable in total cell lysates from all six tested cell lines (Figure 2A). Relatively high IL-4 protein levels were found in lysates prepared from COLO-357 and MIA PaCa-2 cells and relatively low IL-4 protein levels were found in lysates prepared from PANC-1 and T3M4 cells (Figure 2A). A subsequent analysis of serum-free medium revealed that IL-4 could also be detected in the supernatant of all six cell lines. Mean±s.d. (pg ml^−1^ 10^−5^ cells) IL-4 protein concentrations in the supernatant were 1.0±0.7, 0.9±0.3, 1.1±0.9, 1.5±0.6, and 2.5±0.2 for ASPC-1, CAPAN-1, COLO-357, MIA PaCa-2, and T3M4 cells, respectively. Relatively high IL-4 concentrations were detectable in PANC-1 cells (5.3±1.7 s.d. (pg ml^−1^ 10^−5^ cells)).

Real-time PCR analysis using specific primers for the IL-4-encoding sequence revealed the presence of IL-4 mRNA transcripts in all six cell lines. Interleukin-4 mRNA levels correlated with IL-4 protein levels after normalisation to *β*-actin used as internal reference gene in four cell lines; however, relatively high IL-4 mRNA levels and relatively low protein levels were found in CAPAN-1 and especially in PANC-1 cells (Figure 2B).

### Effects of neutralising IL-4 antibodies on cell growth

**Figure 3:**
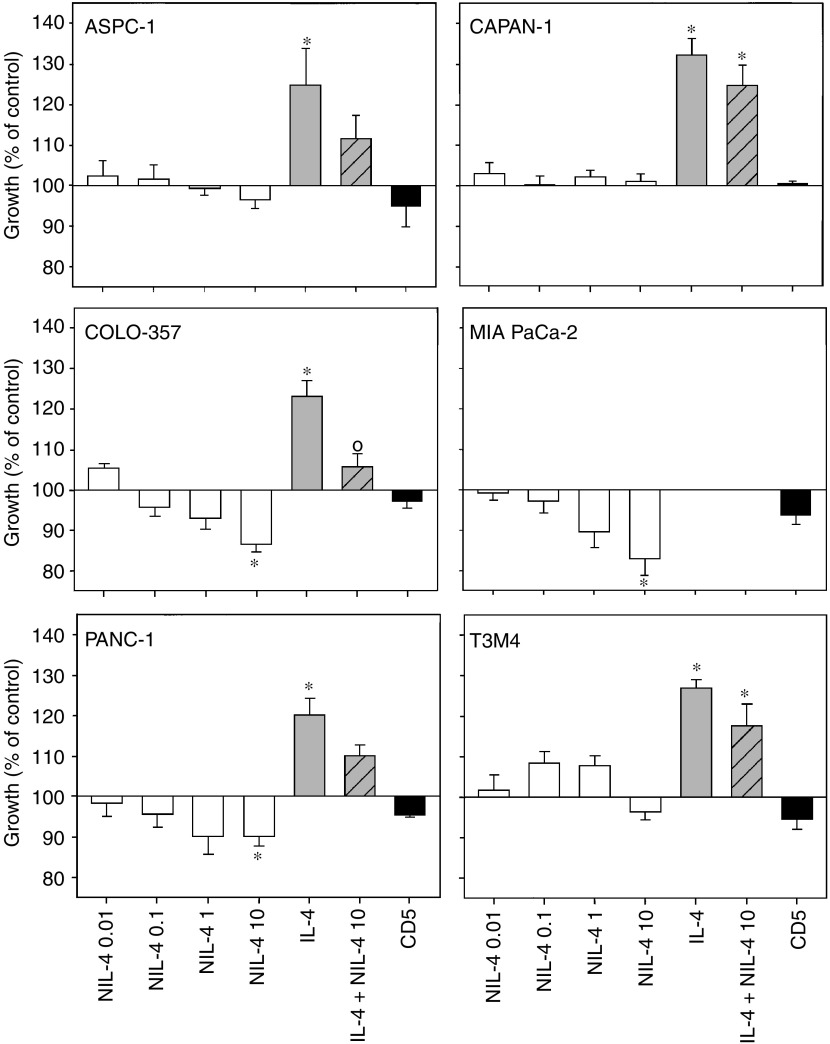
Effect of IL-4 and neutralising IL-4 antibodies (NIL-4) on pancreatic cancer cell growth. Indicated cells were seeded in 96-well plates and incubated in the absence or presence of increasing concentrations of IL-4 neutralising antibodies (*μ*g) alone, IL-4 (5 nM), or the combination of NIL-4 (10 *μ*g) and IL-4 (5 nM). Cell growth was assessed after 48 h by MTT assay. Results are expressed as growth in % of the corresponding untreated controls and are the means (±s.e.m.) of quadruplicate determinations from at least three separate experiments. (^*^) *P*<0.05 compared to untreated controls. (°) *P*<0.05 compared to IL-4-treated cells.

To determine whether there is a potential for IL-4-mediated autocrine growth stimulation, cells were incubated with increasing concentrations of an IL-4 neutralising antibody. The basal growth of COLO-357 and PANC-1 cells was slightly inhibited in a dose-dependent manner, with maximal inhibition of 13% (±2.0% s.e.m.) and 10% (±2.4% s.e.m.), respectively, occurring at 10 *μ*g ml^−1^ of the anti-IL-4 antibody (Figure 3). The effects were minor, but reproducible (*n*=7 for PANC-1 and *n*=10 for COLO-357). Interestingly, the basal growth of IL-4-unresponsive MIA PaCa-2 cells, which express high endogenous IL-4 levels, was also inhibited in the presence of IL-4 neutralising antibodies by 17% (±4.19% s.e.m.). A CD5 control antibody (10 *μ*g ml^−1^) only slightly altered the growth of the six cell lines.

In order to test the specificity of this antibody, the effects of the IL-4 neutralising antibody on cell growth in the presence of exogenous IL-4 were examined. Interleukin-4-stimulated growth (5 nM) of COLO-357 cells was significantly inhibited by incubation with 10 *μ*g ml^−1^ of neutralising IL-4 antibody. This antibody also attenuated the effects of exogenous IL-4 (5 nM) in the other tested cell lines; however, the effect did not reach significance (Figure 3), demonstrating that this antibody can at least partially block IL-4-mediated proliferative effects.

### Effect of IL-4 on signal transduction

**Figure 4:**
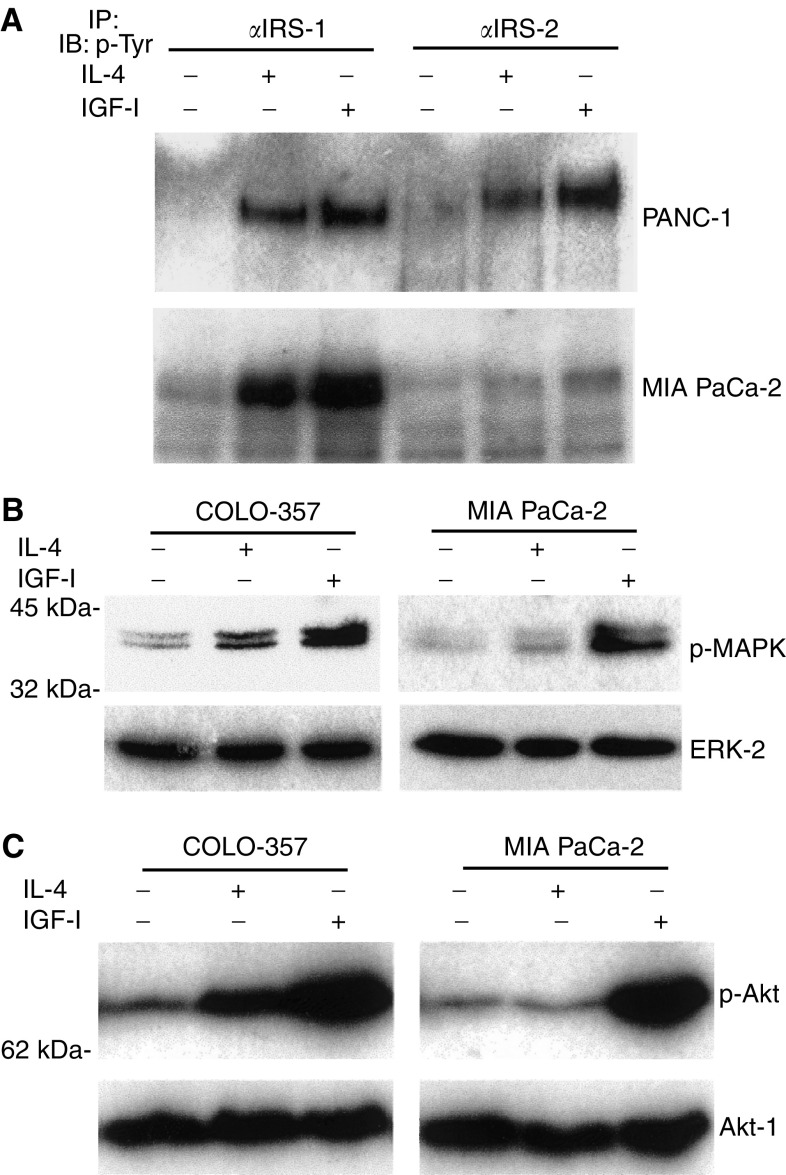
Effect of IL-4 and IGF-I on IRS tyrosine phosphorylation, and MAPK and Akt activation. Indicated cells were serum starved for 18 h before the respective treatment in the absence (−) or presence (+) of IL-4 (5 nM) or IGF-I (5 nM) for 5 min. (**A**) IRS-1 and IRS-2 tyrosine phosphorylation. Immunoprecipitation (IP) of total cell lysates (1 mg ml^−1^) was carried out with IRS-1 or IRS-2 antibodies (4 *μ*g sample^−1^), followed by immunoblot analysis (IB) using a phosphor-tyrosine antibody (p-Tyr). (**B**) Mitogen-activated protein kinase and (**C**) Akt activation in IL-4-responsive COLO-357 and IL-4-nonresponsive MIA PaCa-2 cells. Immunoblot analysis of total cell lysates was carried out with specific antibodies detecting phosphorylated forms of ERK-1 and ERK-2 (p-MAPK, **B**) or Akt-1 (p-Akt, **C**) in each upper panel. To confirm specificity and equal loading (lower panels), membranes were stripped and reprobed with a pan-ERK-2 antibody (ERK-2, **B**) or a pan-Akt-1 antibody (Akt-1, **C**). Molecular weight markers are indicated on the left.

Intracellular signalling of IL-4 is mediated via Janus kinases (JAKs), cytoplasmic protein tyrosine kinases that associate with the receptor complex and phosphorylate the receptor as well as other substrates recruited to the receptor complex. First, the IL-4-dependent tyrosine phosphorylation of IRS-1 and -2, overexpressed in human pancreatic cancer, was analysed. Similar to IGF-I, IL-4 (5 nM for 5 min) induced tyrosine phosphorylation of IRS-1 and IRS-2 in PANC-1 (Figure 4A) and the other four IL-4-responsive cell lines (not shown). It also induced tyrosine phosphorylation of IRS-1 in MIA PaCa-2 cells unresponsive to exogenous IL-4 (Figure 4A). In MIA PaCa-2 cells, which express only low levels of IRS-2 (Kornmann *et al*, 1998), no clear enhancement of IRS-2 tyrosine phosphorylation could be demonstrated.

Mitogen-activated protein kinases mediate mitogenic signalling of several growth factors including IGF-I and cytokines. Both IL-4 and IGF-I enhanced MAPK activity in IL-4-responsive COLO-357 cells (Figure 4B). This effect was less pronounced for IL-4 in comparison to IGF-I, but reproducible (*n*=3), and was also observed in the other IL-4-responsive cell lines (not shown). In contrast, in IL-4-nonresponsive MIA PaCa-2 cells, IGF-I, but not IL-4, enhanced MAPK activity (Figure 4B).

Akt is activated by insulin, various growth factors, and survival factors, and functions in a wortmannin-sensitive pathway involving PI-3 kinase (Franke *et al*, 1997). Both IL-4 and IGF-I enhanced Akt activity in IL-4-responsive COLO-357 cells (Figure 4C) and in the other IL-4-responsive cell lines (not shown). Also the activation of Akt was less pronounced for IL-4 in comparison to IGF-I, but reproducible (*n*=3). In IL-4-nonresponsive MIA PaCa-2 cells, IGF-I, but not IL-4, enhanced Akt activity (Figure 4C).

### Expression and activation of Stat by IL-4

**Figure 5:**
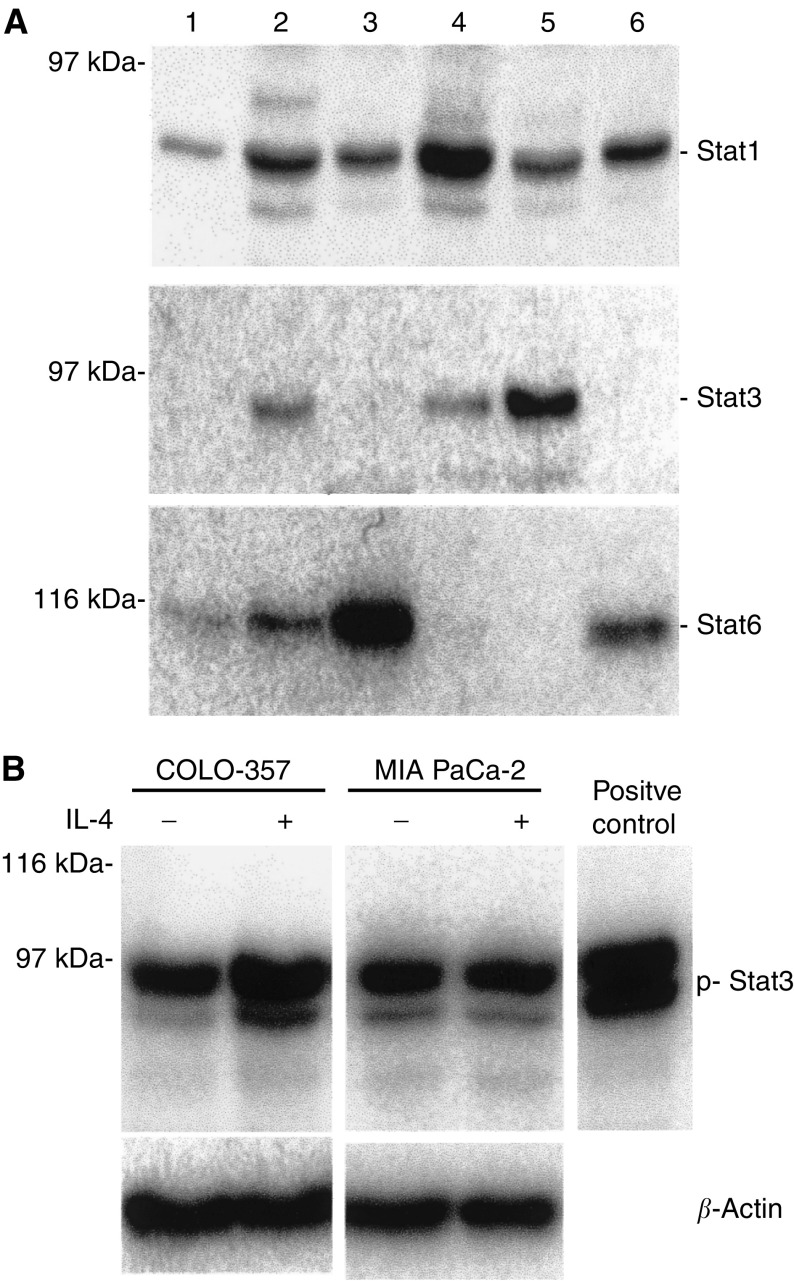
Expression of Stat protein and IL-4-induced Stat phosphorylation in pancreatic cancer cells. (**A**) Stat1, 3, and 6 expression. Immunoblot analysis of total cell lysates was carried out with specific antibodies detecting indicated Stat proteins. (1) ASPC-1, (2) CAPAN-1, (3) COLO-357, (4) MIA PaCa-2, (5) PANC-1, (6) T3M4 cells. (**B**) Effects of IL-4 on Stat3 phosphorylation. Indicated cells were serum starved for 18 h before treatment in the absence (−) or presence (+) of IL-4 (5 nM) for 5 min. Immunoblot analysis of total cell lysates was carried out with specific antibodies detecting the phosphorylated forms of Stat3. A sample with known Stat3 phosphorylation was used as a positive control. *β*-Actin served as a loading control (lower panel). Molecular weight markers are indicated on the left.

In addition to IRS-1 and IRS-2, IL-4 has been reported to be able to activate the transcription factors Stat1, Stat3, and Stat6. At first, we examined whether pancreatic cancer cell lines express Stat1, Stat3, and Stat6. The Stat1 protein was expressed in all six cell lines at various levels (Figure 5A). Highest levels of Stat1 (85 kDa) were observed in MIA PaCa-2 cells. The Stat3 immunoblot analysis revealed a band of 92 kDa readily visible in CAPAN-1, MIA PaCa-2, and PANC-1 cells corresponding to the Stat3*α* isoform. A very faint Stat3 band was also detectable in the other cell lines after a long exposure; Stat6 (120 kDa) was also expressed in pancreatic cancer cells at various levels, with the highest levels found in COLO-357 cells (Figure 5A).

Phosphorylation of Stat occurs in response to a broad spectrum of physiologic stimuli, including activation of cytokine receptors and receptor-tyrosine kinases. Next, we examined activation of Stat1, Stat3, and Stat6 after incubation with IL-4 by immunoblotting using phospho-specific Stat antibodies. Interleukin-4 enhanced Stat3 phosphorylation in IL-4-responsive COLO-357 cells (Figure 5B) and other IL-4-responsive cells (not shown), but not in IL-4-nonresponsive MIA PaCa-2 cells (Figure 5B). We did not observe an increase of Stat1 or Stat6 phosphorylation on incubating any of the cell lines with IL-4 even after long exposure and using positive phosphorylation controls provided by the manufacturer (not shown).

## DISCUSSION

Interleukin-4 is an anti-inflammatory cytokine involved in various immune responses (Nelms *et al*, 1999). Nevertheless, in addition to its expression and functions in immune cells, IL-4R*α* receptors were found to be widely expressed on solid human tumours including pancreatic cancer (Kawakami *et al*, 2001). Initially, it was hypothesised on the basis of upregulation of IL-4R molecules in malignant tumours of epithelial origin that IL-4R may be a product of an oncogene that could be involved in the process of carcinogenesis (Al Jabaari *et al*, 1989). However, in most of the examined tumour cell lines, IL-4 induced growth inhibition (Hoon *et al*, 1991; Gallagher and Zaloom, 1992; Morisaki *et al*, 1992; Toi *et al*, 1992, Obiri *et al*, 1993). Only in Burkitt's lymphoma (Chang *et al*, 2000), in three of 35 malignant B-samples (Taylor *et al*, 1990), and in human head and neck squamous cell carcinoma cells (Myers *et al*, 1996), IL-4 was reported to stimulate cell proliferation. The present study revealed for the first time that IL-4 also exerts proliferative effects in the majority of pancreatic cancer cell lines. Flow cytometry and cell counting in comparison to the potent mitogenic growth factor IGF-I revealed that IL-4 is less potent, but exerts effects similar to IGF-I in increasing cell number and S-phase fraction.

Myers *et al* (1996) proposed a possible explanation for the disparity in the observed effects of IL-4 on the growth of tumour cell lines. Various IL-4R-expressing tumour types may have different downstream mediators of IL-4 action. To date, a number of cytoplasmic signalling proteins have been shown to be phosphorylated in response to IL-4R stimulation, including Jak1, Stat1, Stat3, Stat6, IRS-2, and others (Zamorano and Keegan, 1998; Nelms *et al*, 1999; Chang *et al*, 2000).

Insulin receptor substrate proteins are large adaptor PTB domain proteins involved in insulin and IGF-1 signalling (White and Yenush, 1998); IRS-2, in particular, was also identified as one of the predominant proteins phosphorylated in response to IL-4 (Sun *et al*, 1995). Recruitment of IRS-2 to the activated IL-4R*α* chain results in its phosphorylation and subsequent activation of downstream signalling proteins, including phosphatidylinositol-3 (PI-3) kinase (Sun *et al*, 1995). We have shown that both IGF-I and IL-4 induced the tyrosine phosphorylation of IRS-1 and IRS-2 in five IL-4-responsive cell lines. Interestingly, IL-4 also induced the tyrosine phosphorylation of IRS-1 in IL-4-unresponsive MIA PaCa-2 cells, demonstrating that the initial IL-4 signal transduction is functional in MIA PaCa-2 cells.

The MAPK and Akt pathways are also key components in the signal transduction of many mitogenic growth factors such as IGF-I and are known to regulate proliferation and apoptosis in different cancers. They are classically activated following ligand–receptor interaction by the sequential activation of a linear cascade of protein kinases, including Ras, Raf-1, MEK, and the MAPKs ERK-1 and ERK-2 (Puri and Siegel, 1993; Smerz-Bertling and Duschl, 1995) on the one hand and PI 3-kinase and Akt on the other hand (Franke *et al*, 1997; Fahy *et al*, 2003). Activation of the MAPK and Akt cascades has been observed for several other cytokines, including IL-3, IL-12, and IL-13. In the present study, we demonstrated for the first time that IL-4 activates MAPK and Akt in IL-4-responsive pancreatic cancer cells. In IL-4-nonresponsive MIA PaCa-2 cells, IGF-I but not IL-4 enhanced MAPK and Akt activity, suggesting that MAPK and Akt activation are essential for IL-4-induced cell proliferation in pancreatic cancer cells.

Interleukin-4 may exert various effects dependent on the activation of specific Stat transcription factors as proposed by Chang *et al* (2000). All human pancreatic cancer cells tested expressed various levels of Stat1, Stat3, and Stat6 proteins. However, IL-4-induced activation was only observed for Stat3 in IL-4-responsive cells similar to MAPK and Akt, while in IL-4-nonresponsive cells, IL-4 did not induce phosphorylation of Stat3. This finding is especially exciting in regard to the recently published results of Scholz *et al* (2003), demonstrating that activated Stat3 can promote the malignant phenotype of human pancreatic cancer by acceleration of G1/S-phase progression and thus supporting our present results.

The unresponsiveness of MIA PaCa-2 cells to exogenous IL-4 remains to be determined. However, there are several possible explanations. First, as shown by Northern blot analysis, MIA PaCa-2 cells express lowest levels of IL-4R*α* (Kornmann *et al*, 1999a). In association with the high endogenous IL-4 levels, this may result in unresponsiveness to exogenous IL-4. This is also supported by the observation that neutralising IL-4 antibodies significantly inhibited the basal growth of MIA PaCa-2 cells. Second, MIA PaCa-2 cells have shown only a slight activation of IRS-2 as compared to the other cell lines resulting in subsequent nonactivation of the MAPK, Akt, and Stat3 pathways. Additional other alternative pathways, however, cannot be ruled out at this point. It is also possible that the expression of the IL-13R*α*1 chain, which associates with the IL-4R*α* chain to form a functional receptor, may influence the effects of exogenous IL-4 although MIA PaCa-2 cells as well as the other pancreatic cancer cells lines express IL-13R*α*1 (Kornmann *et al*, 1999a).

Our present study not only demonstrated that IL-4 can exert growth stimulatory effects in pancreatic cancer cells but that they themselves express different amounts of IL-4. To our knowledge, this is a novel finding, which has thus far only been demonstrated in lymphoma cells (Taylor *et al*, 1990; Ford *et al*, 1993). Several lines of evidence in our study suggest that IL-4 has the potential to exert autocrine growth stimulatory effects. First, IL-4 stimulated growth of five pancreatic cancer cell lines. Second, pancreatic cancer cells expressed IL-4. Third, anti-IL-4 neutralising antibodies partially inhibited basal and IL-4-stimulated growth. Together with the observation that pancreatic cancer cells express high levels of IL-4Rs (Kornmann *et al*, 1999a), our results raise the possibility that IL-4 may have a dual effect in human pancreatic cancers. First, IL-4 may act as an autocrine growth factor in pancreatic cancer cells. Second, cancer cell-derived IL-4 may exert paracrine functions on surrounding infiltrating immune cells, inhibiting immune responses.

Overall, our observations may help to better understand the pathobiology of pancreatic cancer in the future giving rise to the possibility that IL-4 and other related cytokines may be important suppressors of cancer-directed immunosurveillance in addition to their growth-promoting effects, thereby facilitating primary pancreatic tumour growth and metastasis. However, to confirm this hypothesis, further studies are necessary.
